# Encoding audio motion: spatial impairment in early blind individuals

**DOI:** 10.3389/fpsyg.2015.01357

**Published:** 2015-09-07

**Authors:** Sara Finocchietti, Giulia Cappagli, Monica Gori

**Affiliations:** Science and Technology for Visually Impaired Children and Adults Group, Istituto Italiano di TecnologiaGenoa, Italy

**Keywords:** auditory perception, blindness, spatial cognition, movement, early blind

## Abstract

The consequence of blindness on auditory spatial localization has been an interesting issue of research in the last decade providing mixed results. Enhanced auditory spatial skills in individuals with visual impairment have been reported by multiple studies, while some aspects of spatial hearing seem to be impaired in the absence of vision. In this study, the ability to encode the trajectory of a 2-dimensional sound motion, reproducing the complete movement, and reaching the correct end-point sound position, is evaluated in 12 early blind (EB) individuals, 8 late blind (LB) individuals, and 20 age-matched sighted blindfolded controls. EB individuals correctly determine the direction of the sound motion on the horizontal axis, but show a clear deficit in encoding the sound motion in the lower side of the plane. On the contrary, LB individuals and blindfolded controls perform much better with no deficit in the lower side of the plane. In fact the mean localization error resulted 271 ± 10 mm for EB individuals, 65 ± 4 mm for LB individuals, and 68 ± 2 mm for sighted blindfolded controls. These results support the hypothesis that (i) it exists a trade-off between the development of enhanced perceptual abilities and role of vision in the sound localization abilities of EB individuals, and (ii) the visual information is fundamental in calibrating some aspects of the representation of auditory space in the brain.

## Introduction

Together with the visual information, audition provides important cues for the perception of object localization and movement. Visual and auditory spatial cues are usually associated. It has been demonstrated that our brain can increase spatial localization precision by integrating these two cues (Stein and Stanford, 2008). However, which is the role of vision on the development of auditory spatial skills is still unclear. Auditory space representation in visually deprived individuals has been extensively studied. The loss of vision results in changes in auditory perceptual abilities and in the way sounds are processed within the brain. An enhancement of certain aspects of spatial hearing and an impairment of some others have been observed in visually impaired individuals (Thinus-Blanc and Gaunet, 1997). The enhanced performance seems to be related to the recruitment of occipital areas deprived of their normal visual inputs (Gougoux et al., 2005; Collignon et al., 2009). Early-blind subjects are properly able to form spatial topographical maps (Tinti et al., 2006; Fortin et al., 2008), express superior auditory pitch discrimination (Gougoux et al., 2004), and can map the auditory environment with superior accuracy (Lessard et al., 1998; Voss et al., 2004). However, neurophysiological studies support the hypothesis of auditory impairment in absence of vision, showing that vision drives the maturation of auditory spatial properties of superior colliculus neurons (King et al., 1988; King, 2009). The superior sound localization accuracy has usually been reported only for peripheral rather than for central regions of space (Röder et al., 1999; Voss et al., 2004) and for monaural testing conditions (Lessard et al., 1998; Gougoux et al., 2005). Furthermore, the localization in the mid-sagittal plane (Zwiers et al., 2001; Lewald, 2002) and the performance of more complex tasks requiring a metric representation of the auditory space (Gori et al., 2010, 2013) tend to be worse in these subjects than in sighted controls.

Importantly, most of these studies investigated spatial skills of blind using static stimuli. In contrast, the dynamic localization of sounds – which requires a continuous encoding in time and space of a moving sound source – has been largely neglected in the literature, with only a few studies investigating it. Poirier et al. (2006) showed that blind individuals can determine both the nature of a sound stimulus (pure tone or complex sound) and the presence or absence of its movement. Lewald (2013) showed that visually deprived individuals were superior in judging the direction of a sound motion on the horizontal direction. These two studies investigate simple aspects of dynamic sound evaluation like its presence and its direction. Both these tasks do not require the presence of a metric representation of space. Since it has been shown that blind individuals results impaired in performing tasks that require a metric representation of the auditory space (Gori et al., 2013), one may expect to find an impairment when a more complex auditory dynamic task, like the capability of blind individuals to completely reproduce a continuous dynamic sound and to determine its end point, is evaluated. While the discrimination of sound direction can be evaluated by comparing the position of the two sounds in a relative way, the reproduction and definition of a sound end point requires the creation of a complex Euclidian map which considers the relationship between sounds positions in space and time [as well as it occurs in the space bisection task, see (Gori et al., 2013)].

For this reason, we studied the ability of early and late blind (EB and LB) individuals and of sighted blindfolded controls, to encode the trajectory of a 2-dimensional sound motion, reproducing the complete movement, and reaching the correct end-point sound position.

We advance the hypothesis that (i) EB, LB, and sighted blindfolded individuals are able to correctly determine the direction of sound motion on the horizontal direction (as previously shown by (Lewald, 2013), but not in the vertical direction; (ii) contrary to LB and sighted blindfolded individuals, EB individuals are impaired in encoding the complete trajectory and in correctly localizing the end-point sound position.

## Materials and Methods

### Subjects

Forty participants have been enrolled in the study: EB (*N* = 12, 7 females; average age 34 ± 11 years old), LB (*N* = 8, 3 females; average age: 33 ± 13 years old), and sighted blindfolded controls (*N* = 20, 11 females; average age: 32 ± 13 years old). All the participants had similar education (at least an Italian high school diploma, indicating 13 years of school). Clinical details regarding the blind participants are presented in **Table 1**. All the EB participants were blind at birth. All the participants had no history of hearing impairment and were right handed. The handedness was defined by the Edinburgh handedness inventory (Oldfield, 1971). The participants provided written informed consent in accordance with the Declaration of Helsinki. The study was approved by the ethics committee of the local health service (*Comitato Etico, ASL3 Genovese, Italy*).

**Table 1:** Clinical details of the early blind (EB) and late blind (LB) participants.

Participant	Age at test	Gender	Pathology	Age complete blindness
**EB**
#EB1	21	Female	Congenital glaucoma and retinal detachment	Birth
#EB2	25	Female	Retinopathy of prematurity	Birth
#EB3	26	Female	Retinopathy of prematurity	Birth
#EB4	20	Female	Congenital Glaucoma	Birth
#EB5	36	Male	Retinopathy of prematurity	Birth
#EB6	37	Female	Retinopathy of prematurity	Birth
#EB7	49	Male	Retinopathy of prematurity	Birth
#EB8	32	Female	Congenital cataract	Birth
#EB9	26	Female	Retinopathy of prematurity	Birth
#EB10	56	Male	Retinopathy of prematurity	Birth
#EB11	56	Male	Congenital Glaucoma	Birth
#EB12	42	Male	Leber amaurosis	Birth
**LB**
#LB1	27	Male	Corneal opacity	17
#LB2	45	Female	Leber amaurosis	40
#LB3	65	Male	Glaucoma	14
#LB4	25	Male	Retinal detachment	22
#LB5	22	Female	Bilateral uveitis	12
#LB6	27	Male	Damage to the optic nerve	10
#LB7	24	Male	Retinal detachment	13
#LB8	37	Female	Exudative retinopathy	14

*The table shows the age at test, the gender, the pathology, and the age since they became completely blind*.

### Set-Up and Protocol

**FIGURE 1:**
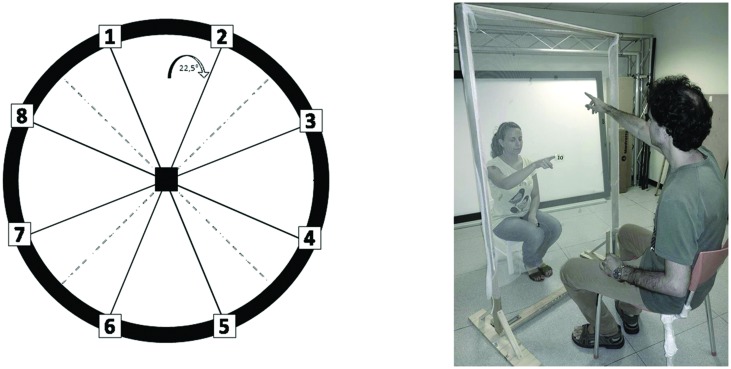
**Experimental set-up**. The graduated circular perimeter (radius = 45 cm) is mounted on a wooden panel positioned in front of the participant on the frontal plane. The eight different positions are marked on the perimeter starting at 22.5° and increasing of 45°.

The experiment was performed in a dark room. The apparatus consisted of a graduated circular perimeter (radius = 45 cm) mounted on a wooden panel positioned in front of the participant on the frontal plane. Eight different positions were marked on the perimeter, starting at 22.5° and increasing of 45° (**Figure 1**). Sighted participants were blindfolded before entering the experimental room. Each participant was seated, the center of the circle corresponding to the tip of his nose, and was able to comfortably reach and explore with their hand the graduated circular perimeter. Two experimenters instructed the participant and performed all the experiments (SF, GC). The two experimenters were previously trained to perform the task as similar as possible, so that the movement’s velocity was consistent across trials, positions, and groups. The experimenter was seated opposite to the participant, holding a sound source. The sound source was a digital metronome (single pulse at 500 Hz, intermittent sound at 180 bpm) and was clearly audible by every participant. A spherical marker was mounted on the distal phalanges of the index finger on both the participant and experimenter for motion tracking (Vicon Motion Systems Ltd., UK). The experimenter moved the sound source from the center of the plane toward one of the possible positions highlighted on the circular perimeter in a randomized order. The participant was instructed to keep his index finger pointed to the center until the end of the audio motion. He then had to reproduce the complete trajectory, reach the estimated sound end-point position, and return to the original central position. The movement was performed at participant’s own pace. All the eight positions were reached five times, for a total of 40 trials per participant.

### Data Analysis

Kinematic data were post-processed and analyzed using Matlab (R2013a, The MathWorks, USA). The spatial accuracy, indicated by localization bias and localization error, was computed for each participant and for each spatial position. Each end-point position was computed as the average of the last 10 samples and normalized on the origin position (the center of the circumference), in order to avoid movement’s errors. The localization bias represents the average position in the space of the end-point reached by the participant. The localization error was calculated as the Euclidean distance (in mm) between the end-point position reached by the participant and the one reached by the experimenter. This error was averaged on the number of trials per position and on the number of participants. In order to evaluate top–bottom and left–right judgments, the end-point positions of the experimenter and the participants were categorized as follows: 1 = top, related to position 1–2, and with ordinate value higher than 0; 2 = right, related to position 3–4, and with abscissae value higher than 0; 3 = bottom, related to position 5–6, and with ordinate value less than 0; 4 = left, related to position 7–8, and with abscissae value less than 0. The correct direction of judgment was defined as the difference between the experimenter and participant categorization was used for further analysis.

### Statistics

Data were normally distributed, confirmed by visual inspection of Q–Q plots. Data are presented as mean and SE. Localization bias was analyzed by two separate factorial ANOVA (one for the abscissae value, one for the ordinate) with factors participant group (EB, LB, controls), and trajectory (experimenter, participant). The Levene’s test for homogeneity of variance was used to compare EB and LB. In order to evaluate the left–right and top–bottom judgments, a factorial ANOVA of the correct direction judgment, with factor participant group (EB, LB, controls), and panel area (top, left, right, bottom) was performed. The localization error was analyzed by a factorial ANOVAs, with between factors participant group (EB, LB, controls), and point (1–8). The mean velocity was analyzed by a one-way ANOVA, with between factor participant group (EB, LB, controls). The Bonferroni *post hoc* test was used in the case of significant factors. *P* < 0.05 was considered significant.

## Results

**FIGURE 2:**
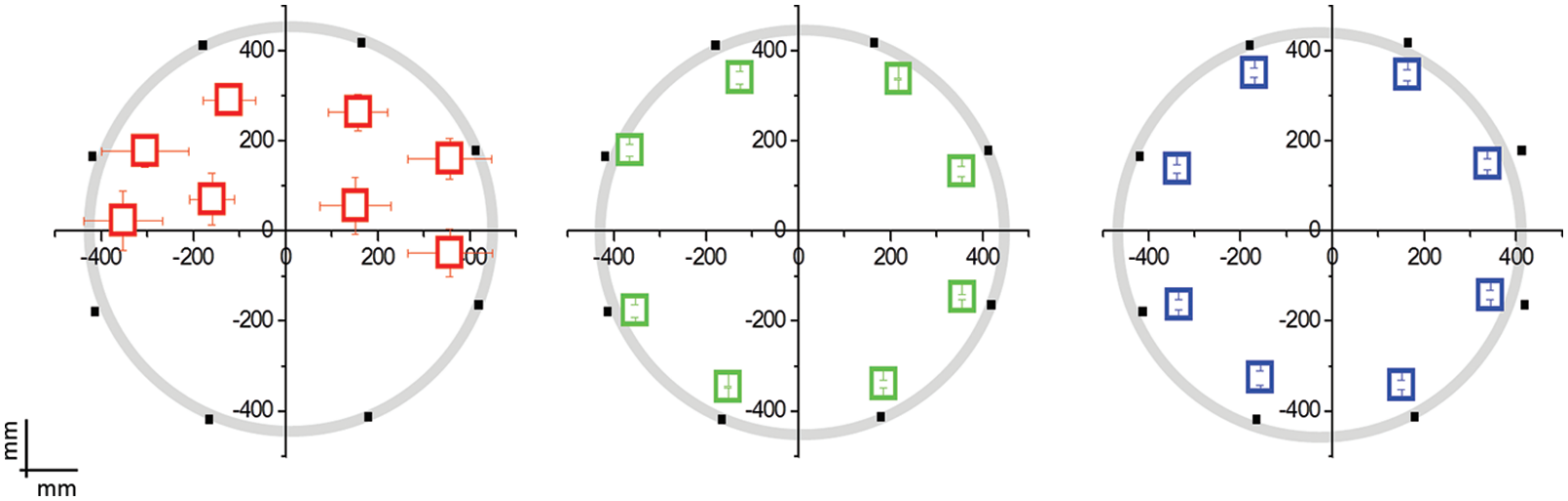
**Mean localization bias in early blind (EB) individuals (*N* = 12; in red), late blind (LB) individuals (*N* = 8; in green), and blindfolded sighted controls (*N* = 20; in blue) relative to the hand pointing task following the moving sound from the origin to one of the eight position on the circle**. The black dots indicate the eight possible end-point positions. The origin (0,0) corresponds to the nose of the participant. EB participants performed far worse than LB individuals or blindfolded controls, presenting a deficit in the lower side positions.

**FIGURE 3:**
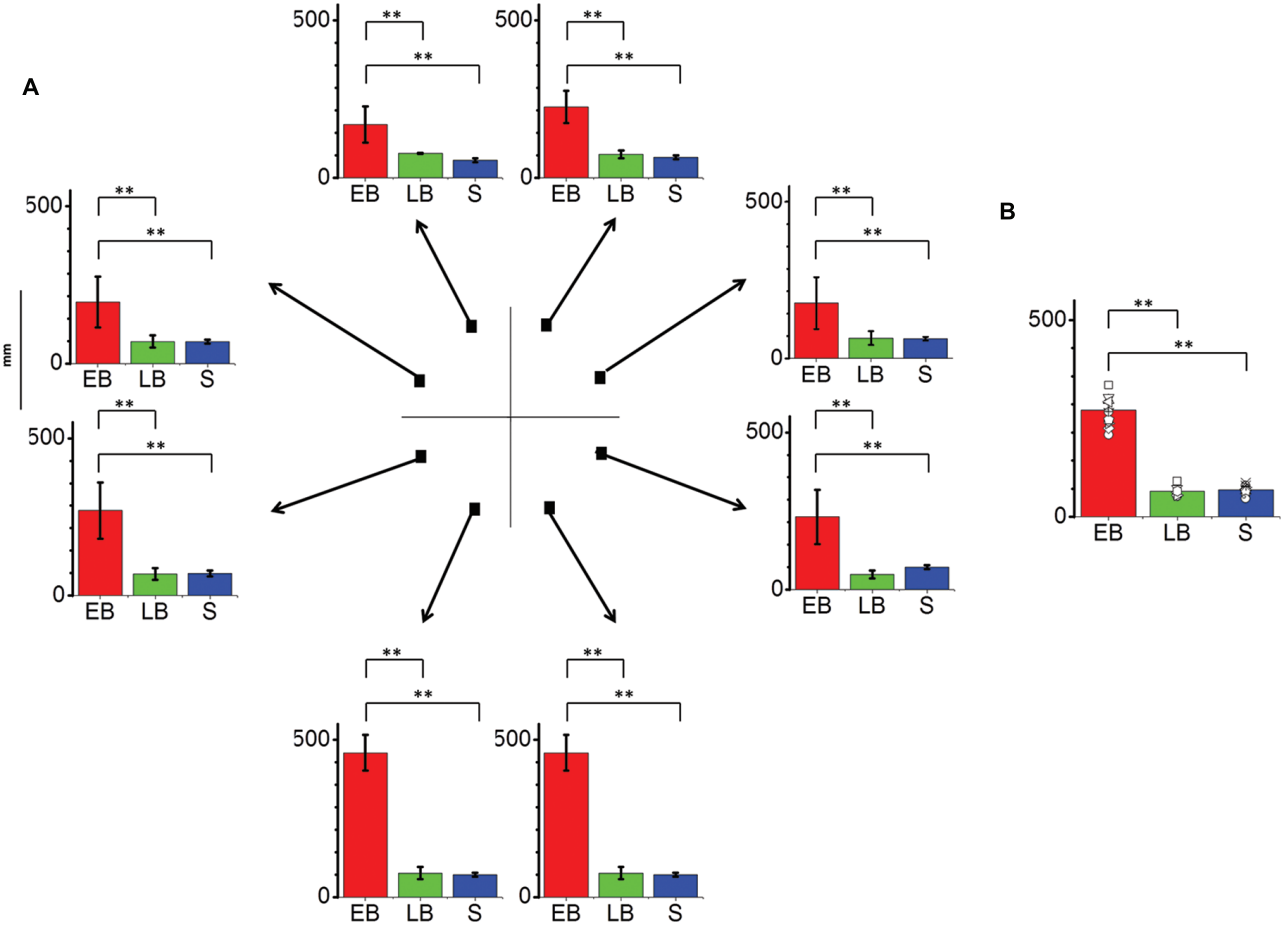
**Significant differences are illustrated (^∗∗^*p* < 0.01). (A)** Average localization error (±SE) for each position and as average across trials for all participants. EB individuals (EB, *N* = 12) perform far worse the task in comparison to LB individuals (LB, *N* = 8) or sighted blindfolded controls (*S, N* = 20), presenting an error over 150 mm in every position and peaking at the lower side positions. **(B)** Average localization error among groups as average across trials and points. EB show an error more than twice bigger than LB and controls.

As can be observed in **Figures 2** and **3**, the pattern of results for the EB is completely different with respect to the ones for the other two groups.

### Localization Bias

The interaction Group × Point resulted significant for both the abscissae (*F*_14,296_ = 5.14; *P* = 0.001) and the ordinate value (*F*_14,296_ = 33.76; *P* = 0.001). LB individuals and sighted individuals do not show any localization bias for both the abscissae and ordinate value (Levene’s: *F*_1,222_ = 0.005; *P* = 0.94): their responses are superimposed with the physical endpoint position (*P* > 0.1; back dots in **Figure 2**). On the contrary, EB individuals showed a strong localization bias and a general compression of the targets toward the upper part of the space (Point 5 and 6, ordinate value, *P* < 0.003).

The interaction Group × Panel area of the correct motion judgment resulted significant (*F*_6,308_ = 24.27; *P* = 0.001). In agreement with previous results (Lewald, 2013), the left/right motion judgment did not show any statistical difference among the three groups (*P* > 0.05), EB individuals, LB individuals and controls were able to correctly judge the stimulus direction in the horizontal axis. On the opposite, the top/bottom motion judgment show statistical difference among the three groups, as EB individuals were not able to correctly judge the stimulus direction in the vertical axis for the bottom positions (*P* < 0.001).

### Localiazion Error

The interaction between group and point resulted significant (*F*_14,296_ = 17.10, *P* = 0.01). In fact the average localization error (**Figure 3**) on lower side positions was more than 400 mm compared to less than 100 mm for both LB individuals and blindfolded controls, respectively. On the opposite, LB individuals performed equal to blindfolded healthy participants, as no statistical difference was present in both localization bias and error (*P* > 0.1).

### Velocity

Every participant was free to perform the movement at his own pace, but no difference in mean velocity between groups was observed (*F*_2,317_ = 0.51; *P* > 0.1).

## Discussion

We present the first study whose aim is to evaluate the dynamic audio localization in visually impaired individuals with a task requiring a continuous encoding in time and space of a sound source in the sagittal plane. This is a complex task that requires the ability to distinguish the spatio-temporal change imposed on moving sounds in space by the dynamic filtering mechanism of the two external ears from the intrinsic spectral structure of the sound (Wightman and Kistler, 1989; Hofman et al., 1998).

Early blind individuals result impaired in performing this task, which results more complex than a static localization task, and they show a clear deficit in encoding the sound motion in the lower side of the plane. On the contrary, LB individuals and blindfolded controls perform much better with no deficit in the lower side of the plane. In agreement with previous results (Lewald, 2013), no deficit was observed in EB subjects for the identification of sound direction.

Some studies suggest that the absence of vision does not impact audio perception in visually impaired humans (Lessard et al., 1998; Röder et al., 1999; Lewald, 2013) and animals (Rauschecker et al., 1995; King and Parsons, 1999). These auditory spatial abilities are more remarkable in peripheral than in central regions of space and in the horizontal plane (King and Parsons, 1999; Röder et al., 1999; Voss et al., 2004). In contrast, localization in the mid-sagittal plane tends to be worse in blind individuals than in sighted controls (Zwiers et al., 2001; Lewald, 2002). A possible explanation about the different static localization ability in the horizontal vs. mid-sagittal plane is that the vertical localization is based primarily on spectral cues that are mainly guided by vision (Tollin et al., 2013). In addition visually impaired individuals, especially EB individuals, show impairments in performing more complex tasks that require a metric representation of space (Gori et al., 2013).

In the first years of life, the brain continuously needs to calibrate the developing system (Gori et al., 2008, 2010; Nardini et al., 2008; Gori, 2015). In case of a sensory loss, such as vision in EB individuals, the important communication between sensory modalities cannot occur (Warren and Pick, 1970) and this can directly affect the development of the audio spatial maps in the superior colliculus (King et al., 1988). While the development of a complex Euclidian representation of space is compromised in absence of vision from birth (Gori et al., 2013), results obtained in LB individuals suggest that even a short early visual experience can guarantee this representation (Fine et al., 2003). Some EB individuals can partly build a representation of space in the case of simple audio spatial tasks, like monaural static sound localization (Röder et al., 1999; Voss et al., 2004), with changes within the auditory pathway and the recruitment of the visual cortex (Merabet and Pascual-Leone, 2009; Bavelier and Hirshorn, 2010). In our case, both early and LB individuals show, in agreement with previous studies (Poirier et al., 2006; Lewald, 2013), an equal auditory motion perception on the horizontal axis. Our task resulted more complex as it required the ability to relate sound positions in a two dimensional space and time; in this case other brain areas cannot intervene, and EB individuals clearly result impaired. What is the reason for this? When the visual calibration is not possible, audio spatial information may be self-calibrated by the auditory system. This audio self-calibration is limited by: (i) the physiology of the auditory system and associated processing of the audio signal; and (ii) the audio environmental statistics.

First, like in the case of the elevation-related spectral cues (Zwiers et al., 2001; Lewald, 2002), the auditory system is not equally good in perceiving sounds coming from the frontal or from the peripheral plane (King, 2009). This suggests a trade-off in the localization proficiency between the two auditory spatial planes that has recently proposed for a static auditory localization task (Voss et al., 2015). The ability to perform such a complex task may then require a full development of the audio spatial maps in superior colliculus, where signals from the different senses are combined and used to guide adaptive motor responses.

Second, in the peri-personal space, the most frequent dynamic sounds we face with are the ones related to individuals speaking around us, sounds that generally are at our height. Recent findings show that the natural auditory scene statistics shapes human spatial hearing, suggesting that both sound localization behavior and ear anatomy are fine-tuned to the statistics of natural auditory scenes (Parise et al., 2014). This statistical environmental cue may then affect the way blind individuals built their spatial representation.

## Conclusion

The absence of spatial references from the visual inputs has widespread consequences on the brain; the important communication between sensory modalities cannot be created, therefore auditory space perception can only rely on the physiological and statistical information heterogeneity. This information results insufficient in dynamic localization tasks, as the one presented here, producing direct impairments on auditory space cognition in blind individuals.

## Conflict of Interest Statement

The authors declare that the research was conducted in the absence of any commercial or financial relationships that could be construed as a potential conflict of interest.

## References

[B1] Bavelier D. Hirshorn E. A. (2010). I see where you’re hearing: how cross-modal plasticity may exploit homologous brain structures. *Nat. Neurosci.* 13 1309–1311. 10.1038/nn1110-1309 20975752

[B2] Collignon O. Voss P. Lassonde M. Lepore F. (2009). Cross-modal plasticity for the spatial processing of sounds in visually deprived subjects. *Exp. Brain Res.* 192 343–358. 10.1007/s00221-008-1553-z 18762928

[B3] Fine I. Wade A. R. Brewer A. A. May M. G. Goodman D. F. Boynton G. M. (2003). Long-term deprivation affects visual perception and cortex. *Nat. Neurosci.* 6 915–916. 10.1038/nn1102 12937420

[B4] Fortin M. Voss P. Lord C. Lassonde M. Pruessner J. Saint-Amour D. (2008). Wayfinding in the blind: larger hippocampal volume and supranormal spatial navigation. *Brain* 131 2995–3005. 10.1093/brain/awn250 18854327

[B5] Gori M. (2015). Multisensory integration and calibration in children and adults with and without sensory and motor disabilities. *Multisens. Res.* 28 71–99. 10.1163/22134808-00002478 26152053

[B6] Gori M. Del Viva M. Sandini G. Burr D. C. (2008). Young children do not integrate visual and haptic form information. *Curr. Biol.* 18 694–698. 10.1016/j.cub.2008.04.036 18450446

[B7] Gori M. Sandini G. Martinoli C. Burr D. (2010). Poor haptic orientation discrimination in nonsighted children may reflect disruption of cross-sensory calibration. *Curr. Biol.* 20 223–225. 10.1016/j.cub.2009.11.069 20116249

[B8] Gori M. Sandini G. Martinoli C. Burr D. C. (2013). Impairment of auditory spatial localization in congenitally blind human subjects. *Brain* 137 288–293. 10.1093/brain/awt311 PMC3891446 24271326

[B9] Gougoux F. Lepore F. Lassonde M. Voss P. Zatorre R. J. Belin P. (2004). Neuropsychology: pitch discrimination in the early blind. *Nature* 430 309 10.1038/430309a 15254527

[B10] Gougoux F. Zatorre R. J. Lassonde M. Voss P. Lepore F. (2005). A functional neuroimaging study of sound localization: visual cortex activity predicts performance in early-blind individuals. *PLoS Biol.* 3:e27 10.1371/journal.pbio.0030027 PMC544927 15678166

[B11] Hofman P. M. Van Riswick J. G. Van Opstal A. J. (1998). Relearning sound localization with new ears. *Nat. Neurosci.* 1 417–421. 10.1038/1633 10196533

[B12] King A. J. (2009). Visual influences on auditory spatial learning. *Philos. Trans. R. Soc. B Biol. Sci.* 364 331–339. 10.1098/rstb.2008.0230 PMC2674475 18986967

[B13] King A. J. Hutchings M. E. Moore D. R. Blakemore C. (1988). Developmental plasticity in the visual and auditory representations in the mammalian superior colliculus. *Nature* 332 73–76. 10.1038/332073a0 3347247

[B14] King A. J. Parsons C. H. (1999). Improved auditory spatial acuity in visually deprived ferrets. *Euro. J. Neurosci.* 11 3945–3956. 10.1046/j.1460-9568.1999.00821.x 10583483

[B15] Lessard N. Pare M. Lepore F. Lassonde M. (1998). Early-blind human subjects localize sound sources better than sighted subjects. *Nature* 395 278–280. 10.1038/26228 9751055

[B16] Lewald J. (2002). Vertical sound localization in blind humans. *Neuropsychologia* 40 1868–1872. 10.1016/S0028-3932(02)00071-4 12207985

[B17] Lewald J. (2013). Exceptional ability of blind humans to hear sound motion: implications for the emergence of auditory space. *Neuropsychologia* 51 181–186. 10.1016/j.neuropsychologia.2012.11.017 23178211

[B18] Merabet L. B. Pascual-Leone A. (2009). Neural reorganization following sensory loss: the opportunity of change. *Nat. Rev. Neurosci.* 11 44–52. 10.1038/nrn2758 PMC3898172 19935836

[B19] Nardini M. Jones P. Bedford R. Braddick O. (2008). Development of cue integration in human navigation. *Curr. Biol.* 18 689–693. 10.1016/j.cub.2008.04.021 18450447

[B20] Oldfield R. C. (1971). The assessment and analysis of handedness: the Edinburgh inventory. *Neuropsychologia* 9 97–113. 10.1016/0028-3932(71)90067-4 5146491

[B21] Parise C. V. Knorre K. Ernst M. O. (2014). Natural auditory scene statistics shapes human spatial hearing. *Proc. Natl. Acad. Sci. U.S.A.* 111 6104–6108. 10.1073/pnas.1322705111 PMC4000839 24711409

[B22] Poirier C. Collignon O. Scheiber C. Renier L. Vanlierde A. Tranduy D. (2006). Auditory motion perception activates visual motion areas in early blind subjects. *Neuroimage* 31 279–285. 10.1016/j.neuroimage.2005.11.036 16443376

[B23] Rauschecker J. P. Tian B. Hauser M. (1995). Processing of complex sounds in the macaque nonprimary auditory cortex. *Science* 268 111–114. 10.1126/science.7701330 7701330

[B24] Röder B. Teder-Sälejärvi W. Sterr A. Rösler F. Hillyard S. A. Neville H. J. (1999). Improved auditory spatial tuning in blind humans. *Nature* 400 162–166. 10.1038/22106 10408442

[B25] Stein B. E. Stanford T. R. (2008). Multisensory integration: current issues from the perspective of the single neuron. *Nat. Rev. Neurosci.* 9 255–266. 10.1038/nrn2331 18354398

[B26] Thinus-Blanc C. Gaunet F. (1997). Representation of space in blind persons: vision as a spatial sense? *Psychol. Bull*. 121 20–42. 10.1037/0033-2909.121.1.20 9064698

[B27] Tinti C. Adenzato M. Tamietto M. Cornoldi C. (2006). Visual experience is not necessary for efficient survey spatial cognition: evidence from blindness. *Q. J. Exp. Psychol.* 59 1306–1328. 10.1080/17470210500214275 16769626

[B28] Tollin D. J. Ruhland J. L. Yin T. C. (2013). The role of spectral composition of sounds on the localization of sound sources by cats. *J. Neurophysiol.* 109 1658–1668. 10.1152/jn.00358.2012 PMC3602938 23274314

[B29] Voss P. Lassonde M. Gougoux F. Fortin M. Guillemot J.-P. Lepore F. (2004). Early-and late-onset blind individuals show supra-normal auditory abilities in far-space. *Curr. Biol.* 14 1734–1738. 10.1016/j.cub.2004.09.051 15458644

[B30] Voss P. Tabry V. Zatorre R. J. (2015). Trade-off in the sound localization abilities of early blind individuals between the horizontal and vertical planes. *J. Neurosci.* 35 6051–6056. 10.1523/JNEUROSCI.4544-14.2015 PMC6605175 25878278

[B31] Warren D. H. Pick H. L. (1970). Intermodality relations in localization in blind and sighted people. *Percept. Psychophys.* 8 430–432. 10.3758/BF03207040

[B32] Wightman F. L. Kistler D. J. (1989). Headphone simulation of free-field listening. I: stimulus synthesis. *J. Acous. Soc. Am.* 85 858–867. 10.1121/1.397557 2926000

[B33] Zwiers M. Van Opstal A. Cruysberg J. (2001). A spatial hearing deficit in early-blind humans. *J. Neurosci.* 21 141–145. 10.1523/JNEUROSCI.21-09-j0002.2001 PMC6762556 11312316

